# A Polarized Light Microscopic Study to Comparatively evaluate Four Remineralizing Agents on Enamel *viz* CPP-ACPF, ReminPro, SHY-NM and Colgate Strong Teeth

**DOI:** 10.5005/jp-journals-10005-1281

**Published:** 2015-04-28

**Authors:** Reshma Rajan, Ramesh Krishnan, Bibin Bhaskaran, Suresh V Kumar

**Affiliations:** Senior Lecturer, Department of Pedodontics and Preventive Dentistry, PSM Dental College, Trichur, Kerala, India; Professor, Department of Pedodontics and Preventive Dentistry Vinayaka Mission’s Sankarachariyar Dental College, Salem Tamil Nadu, India; Senior Lecturer, Department of Prosthodontics and Implantology, PSM Dental College, Trichur, Kerala, India; Head, Department of Pedodontics and Preventive Dentistry Vinayaka Mission’s Sankarachariyar Dental College, Salem Tamil Nadu, India

**Keywords:** Demineralization, Bioactive glass, Hydroxyapatite, CPP-ACPF, Polarized light microscope.

## Abstract

**Aim:** To compare and evaluate the remineralizing potential of four commercially available products namely SHY-NM, GC Tooth Mousse Plus, ReminPro and Colgate strong teeth on demineralized human teeth.

**Materials and methods:** The study included 50 extracted premolars having 3 × 3 mm window prepared on the middle third of the tooth, which was then subjected to demineralization for 48 hours at 37°C. Teeth were randomly selected and grouped into five study groups of 10 teeth in each. Each group was treated with respective remineralizing agent and sectioned using hard-tissue microtome. Each section obtained was visualized under polarized light microscope and analyzed using Image J software.

**Results:** The statistically evaluated results revealed that SHY-NM has the most remineralizing potential followed by ReminPro, GC Tooth Mousse Plus and fluoridated toothpaste.

**Conclusion:** Based on the study, the SHY-NM was superior to the GC Tooth Mousse Plus, ReminPro and Colgate strong teeth on demineralized human teeth.

**How to cite this article:** Rajan R, Krishnan R, Bhaskaran B, Kumar SV. A Polarized Light Microscopic Study to Comparatively evaluate Four Remineralizing Agents on Enamel *viz* CPP-ACPF, ReminPro, SHY-NM and Colgate Strong Teeth. Int J Clin Pediatr Dent 2015;8(1):42-47.

## INTRODUCTION

Dental caries is a highly prevalent multifactorial disease, and although in most developed countries, its prevalence has declined, the disease remains a major public health problem in developing countries.

The signs of the caries process cover a continuum from the first molecular changes in the apatite crystals of the tooth, to a visible white-spot lesion, through to dentin involvement and eventual cavitation. Progression through these stages requires a continual imbalance between pathological and protective factors that results in the dissolution of apatite crystals. The first evident clinical sign of dental caries is the white spot lesion, which is vulnerable to acid attack due to loss of carbonate and magnesium.^1^

For many years fluorides have been used for caries prevention and also for remineralization of tooth structure. The major shortcoming of currently available toothpastes, mouth rinses and topical applications is the fact that their ability to remineralize enamel is limited by the low concentration of calcium and phosphate ions available in saliva. This has led to the research of many new materials which can provide the oral environment with the essential elements for remineralization. Some of them are bioactive glass, casein phosphopeptide-amorphous calcium phosphate (CPP-ACP) and hydro-xyapatite with fluoride, etc.^1^

The nanocomplexes of CPP-ACP was derived from bovine milk protein, casein, calcium and phosphate. The anticariogenic mechanism of CPP-ACP is achieved by the incorporation of amorphous calcium phosphate into plaque and onto the tooth surface.^2^

In ReminPro, the hydroxyapatite helps to fill super-ficial enamel lesions and the tiniest irregularities, and fluoride (1,450 ppm) act by strengthening the tooth and making it more resistant to acid attack, thereby helping in natural remineralization.^3^

The bioactive glass which is a biomimetic minera-lizer, releases sodium, calcium and phosphorous ions into the saliva when it comes in contact with oral fluids or water, thereby remineralizing the tooth surface.^4^ It is considered as a breakthrough in remineralization technology, because the current standard treatment for tooth remineralization and prevention of decay is slow acting and is dependent on adequate saliva as a source of calcium and phosphorus.

Thus, the present study was undertaken to evaluate the remineralizing potential of SHY-NM (bioactive glass) GC Tooth Mousse Plus (CPP-ACPF), ReminPro (hydroxy apatite with fluoride and xylitol) and fluoridated toothpaste (fluoride 1000 ppm) using polarized light microscope, and to quantify the remineralized areas by using Image J software.

## MATERIALS AND METHODS

**Fig. 1:**
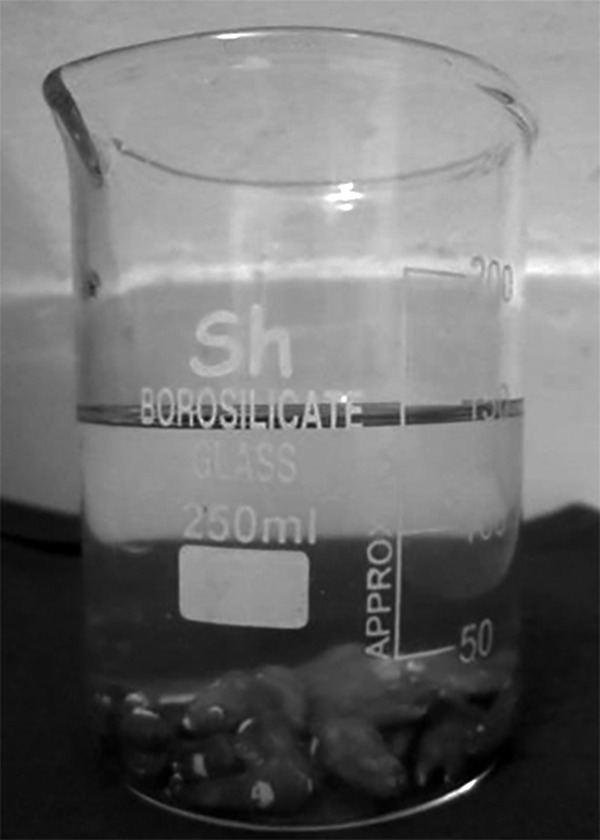
Tooth in demineralization solution

**Fig. 2:**
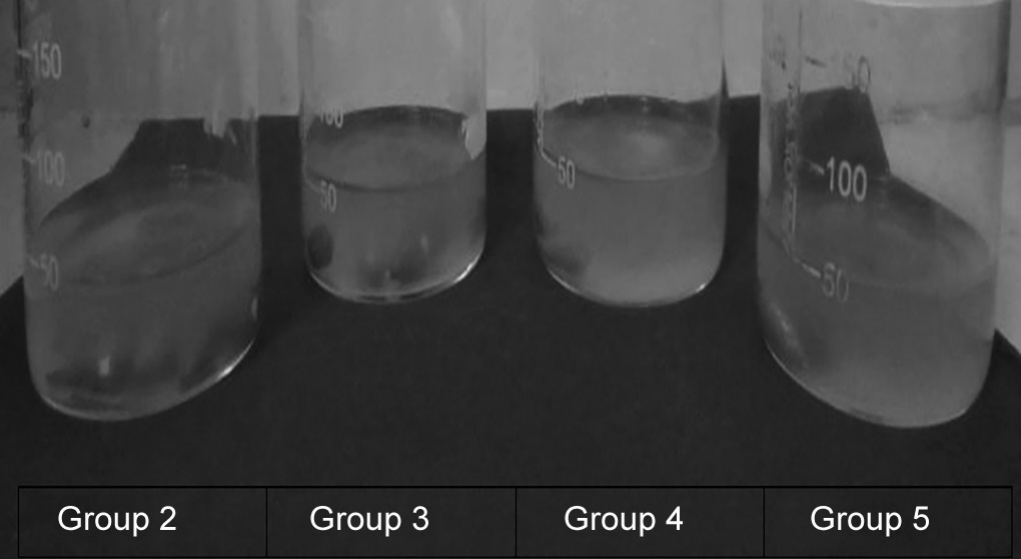
Randomly divided four experimental groups

A 50 sound permanent premolars extracted for orthodontic treatment were collected and stored in 10% formalin. The exclusion criteria include tooth with dental caries, developmental defects, enamel fractures, discoloration, internal/external pathologic resorption, restoration and tooth attempted for pulp therapy. In all the teeth a 3 × 3 mm window was prepared on the middle third of the buccal surface, by sticking adhesive tape and covering the remaining surface with two coats of acid resistant nail varnish. The study samples were then subjected to demineralization by immersing in artificially prepared demineralizing solution (Fig. 1) with a final pH of 4.5 for 48 hours at 37°C. They were randomly divided into the following groups with 10 samples each (Fig. 2):

- Group 1: Demineralized group as negative control (10).
- Group 2: Group treated with fluoridated toothpaste (10).
- Group 3: Group treated with CPP-ACPF (10).
- Group 4: Group treated with ReminPro (10).
- Group 5: Group treated with SHY-NM (10).

**Fig. 3:**
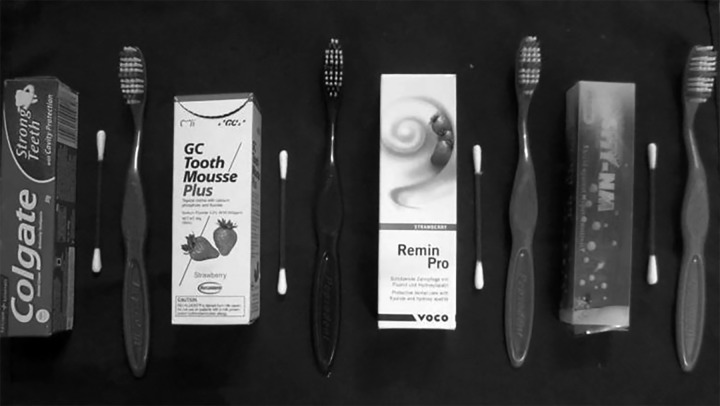
Remineralizing agents

**Fig. 4:**
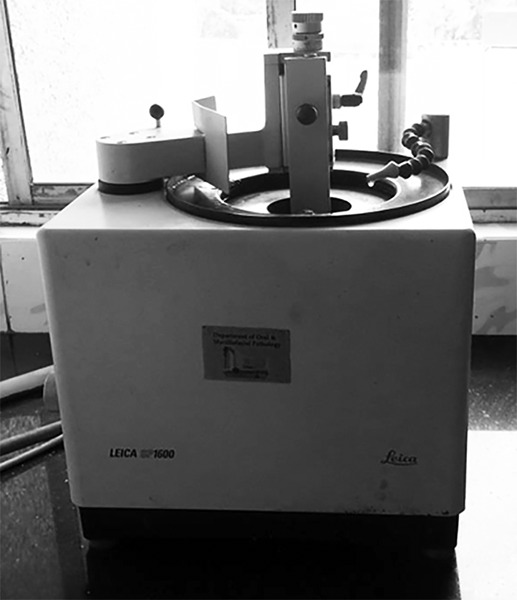
Hard tissue microtome

**Fig. 5:**
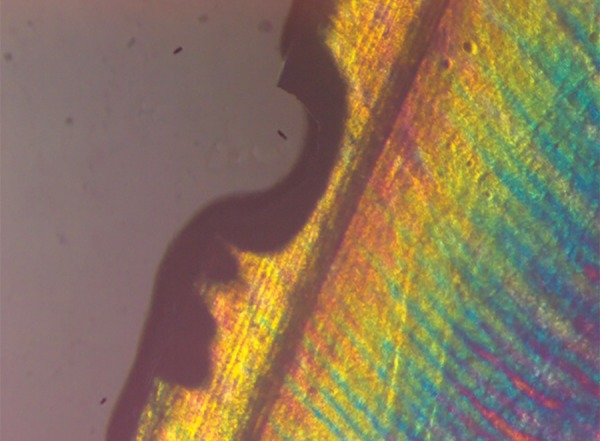
Polarized light microscopy image of representative lesion from the demineralized group

**Fig. 6:**
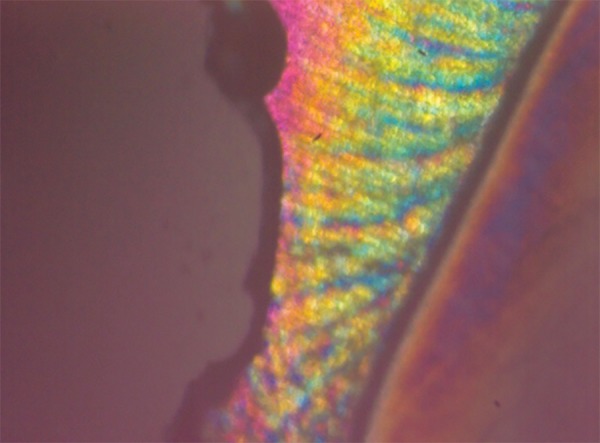
Polarized light microscopy image of representative lesion from the group treated with fluoridated toothpaste

**Fig. 7:**
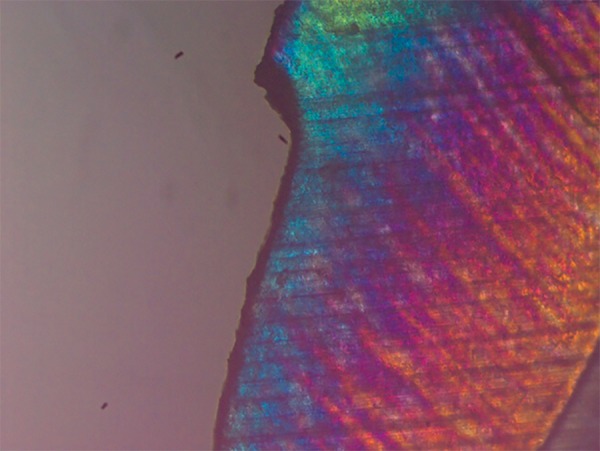
Polarized light microscopy image of representative lesion from the group treated with CPP-ACPF

**Fig. 8:**
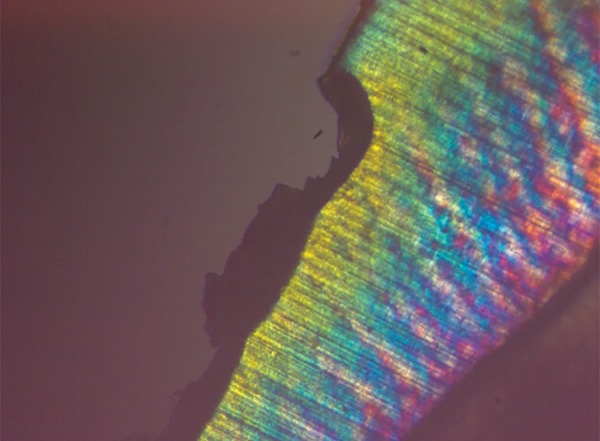
Polarized light microscopy image of representative lesion from the group treated with ReminPro

**Fig. 9:**
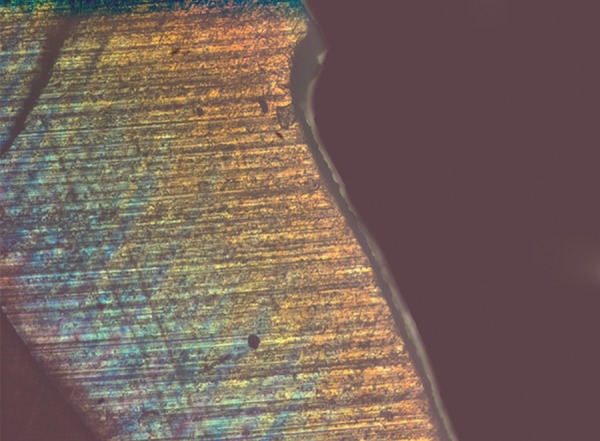
Polarized light microscopy image of representative lesion from the group treated with SHY-NM

**Fig. 10:**
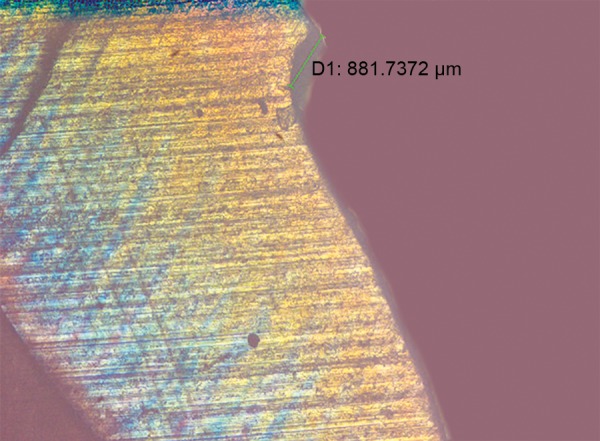
Polarized light microscopy image showing image depth analysis using Image J software

Each group was subjected for remineralization twice daily with respective agents for 4 minutes, once in the morning and later in the afternoon, for 20 consecutive days using cotton applicator (Fig. 3). After each application, all the teeth were cleaned with toothbrush under deionized water and were stored in artificial saliva, which was changed every 48 hours. After the experimental period, all teeth were sectioned using hard tissue microtome (Leica SP 1600) into 200 μm thickness (Fig. 4). Each section obtained were analyzed for depth of the lesion under polarized light microscope (Olympus BX51) (Figs 5 to 9). The lesion depth was measured from the surface of the tooth to the maximum depth using the Image J software (Fig. 10) (Java-based image processing program). The mean and standard deviation (SD) of the lesion depth from each group was obtained and statistical analysis was done using ANOVA and post-hoc test.

## RESULTS

**Graph 1:**
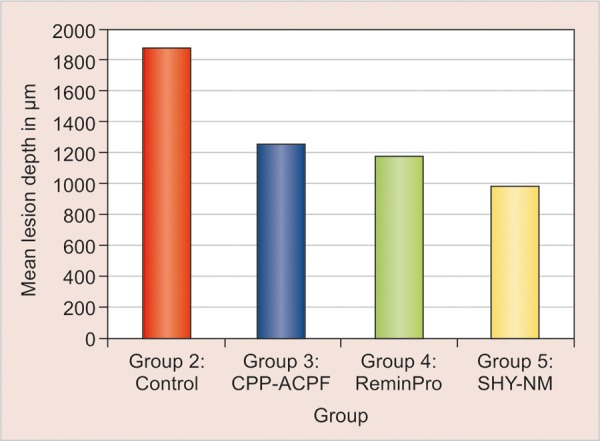
Comparing the mean lesion depth after remineralization

The mean lesion depth of the specimens, ranged from 987.12 to 1880.15 μm and the SD ranged from 149.20 to 502.05 μm. Table 1 shows the intergroup comparison of the mean and SD values of group 2 (1880.15 μm), group 3 (1261.80 μm), group 4 (1185.14 μm) and group 5 (987.12 μm). The p-value obtained was highly significant at 1% level. SHY-NM group illustrates the least mean score (987.12 μm) followed by ReminPro (1185.14 μm), CPP-ACPF (1261.80 μm) and fluoridated toothpaste (1880.15 μm) shown in Graph 1.

Tukey B test was applied to obtain the homogenous subset. The first subset comprises of statistically significant groups which includes CPP-ACPF (1261.80 μm), ReminPro (1185.14 μm) and SHY-NM (987.12 μm) and second subset comprised of fluoridated toothpaste (1880.15 μm) which showed no statistical significance. Post-hoc range test (Table 2) showed the least mean score for SHY-NM (987.12 μm) and the highest mean score for fluoridated toothpaste (1880.15 μm). Thus, the study suggest that SHY-NM has the most remineralizing potential followed by ReminPro, CPP-ACPF and fluoridated toothpaste.

## DISCUSSION

The basic mechanism of remineralization involves the diffusion of calcium and phosphate ions from saliva and other topical sources to build a hypermineralized, acid-resistant, fluorapatite like layer on the existing crystal remnants which act as remineralization nuclei.

Remineralization of white-spot lesions may be possible with a variety of currently available agents, such as fluoride, casein phosphopeptide amorphous calcium phosphate (CPP-ACP) and bioavailable calcium phosphate. This concept bridges the traditional gap between prevention and surgical procedures.^5^

This *in vitro* study was undertaken to compare the remineralizing potential of four commercially available products namely SHY-NM, GC Tooth Mousse Plus, ReminPro and Colgate strong teeth on demineralized human teeth. These four remineralizing agent were chosen based on their difference in composition. SHY-NM contains bioactive glass, GC Tooth Mousse Plus contains CPP-ACP and fluoride (900 ppm), ReminPro contains hydroxyapatite, fluoride (1450 ppm) and xylitol, and fluoridated toothpaste contains 1000 ppm of fluoride.

In this study, the extracted teeth were stored in 10% formalin, because it resists demineralization by fixing proteins present in the organic pellicle attached to the surface of teeth and also act as a best disinfecting agent.^6^ A3 × 3 mm window was prepared on the buccal surface of all the teeth and then the demineralization of the teeth was carried out by immersing the samples in a glass container containing prepared demineralizing solution (50 ml), for a period of 48 hours, at 37°C. The teeth were then randomly divided into groups, treated with respective agents, and were stored in artificial saliva. Each tooth was sectioned to 200 μm thickness using a hard-tissue microtome by cutting through the center of the enamel window.^7^ Polarized light microscope was used to assess the lesion depth because the histological features of dentin and enamel can be visualized better due to its birefringence property, which is not well appreciated in a transmitted light microscope.^8^ Image J software, a Java-based image processing program was used to interpret the lesion depth.^9^ Statistical analysis was done by ANOVA and post-hoc test.^2^

In the present study, SHY-NM group containing bio-active glass (BAG) of 5% calcium sodium phosphosilicate with a particle size of 5 μm, showed a highly significant amount of remineralization. The remineralization observed in this group could be attributed to the sustained and prolonged release of Ca^+^ and P^-^ ions, thus increasing the concentration of available calcium and phosphate for the remineralization of artificially-created carious lesions.^10^

The physical occlusion of bioactive particles begins when the material is subjected to an aqueous environment, as reported by Hench LL et al (1993).^11^ Sodium ions (Na^+^) in the particles immediately begin to exchange with hydrogen cations (H^+^ or H_3_O^+^). This rapid release of ions allows calcium (Ca^+^) ions in the particle structure, as well as phosphate (PO_4_^3-^) ions to be released from the material.^6^ According to Elizabeta Setal (2010),^8^ a localized transient increase in pH occurs, which helps to precipitate the calcium and phosphate ions from the bioactive glass particle, along with the calcium and phosphorus found in saliva, to form a calcium phosphate (Ca-P) layer.

**Table 1:** **Table 1:** Intergroup comparison of lesion depth after remineralization with fluoridated toothpaste, CPP-ACPF, ReminPro and SHY-NM

*Groups*		*N*		*Mean lesion depth in μm*		*SD*		*F*		*p-value*		*Remark*	
Fluoridated toothpaste		10		1,880.15		502.05							
CPP-ACPF		10		1,261.80		390.17							
ReminPro		10		1,185.14		371.47		10.53		<0.001**		Significant at 1%	
SHY-NM		10		987.12		149.20							
Total		40		1,328.55		494.57							

**Highly significant

**Table 2:** **Table 2:** Post-hoc tests–homogeneous subsets

*Tukey B*							
				*Subset for alpha = 0.05*			
*Group*		*N*		*1*		*2*	
SHY-NM		10		987.12 μm			
ReminPro		10		1,185.14 μm			
CPP-ACPF		10		1,261.80 μm			
Fluoridated toothpaste		10				1,880.15 μm	

Following SHY-NM, the other most effective material found in the study was ReminPro, which consisted of hydroxyapatite, fluoride and xylitol. The hydroxyapatite present in this agent fills superficial enamel lesions and the tiniest irregularities that arise from surface deminera-lization. It adheres to the tooth substance thus protecting the tooth against demineralization and it also impairs the adhesion of bacterial plaque. Fluoride (1450 ppm) present in this agent is converted to more stable and more acid-resistant fluorapatite on tooth surface, thus making tooth more resistant to acid attacks. Xylitol–the third component in ReminPro–cannot be converted into harmful lactic acid by cariogenic bacteria. Thus, the harmful effects of these bacteria and its metabolic product can be significantly reduced allowing the mouth to naturally remineralize damaged tooth structure with less interruption. A study done by Marchetti E et al (2011)^12^ demonstrated that when ReminPro was used after bleaching procedure, there was a continuous increase in mineral gain and the return of the microhardness values to near baseline level.

The third remineralizing agent following SHY-NM and ReminPro to show significant effect was a fluoride enriched CPP-ACP formulation–the CPP-ACPF.

Casein phosphopeptide-amorphous calcium phosphate is a nanocomplex derived from bovine milk protein, casein. The casein phosphopeptides (CPP) act as an ACP carrier localizing the highly soluble calcium phosphate phase on tooth. Incorporation of fluoride with CPP-ACP helps in localizing calcium and phosphate with fluoride ions at the tooth surface as CPP-ACPF nanocomplexes. This increased concentration of calcium, phosphate and fluoride ions on the tooth surface leads to the diffusion of ions into the enamel and the subsurface lesion, resulting in higher levels of remineralization and fluoride incorporation into the mineral phase.^2^

Agnihotri et al (2011)^13^ noted that when CPP-ACP was used in combination with fluoride, it lowered caries score and had better effect on inhibiting dem-ineralization of sound enamel. Ogaard et al (1988)^14^ reported that CPP-ACPF containing low fluoride concentration (0.2% or 900 ppm of NaF) maintains a state of supersaturation, and act as an excellent local slow-delivery system to treat the white spot lesions.

The beneficial remineralizing effects of bioactive glass is most likely due to the Ca^2+^, PO_4_^3–^ ions at the tooth surface, and deposition of a mineral layer on dentin that will occlude dentinal tubules and resist demineralization. Thus, the results obtained from this study were similar to those reported by Dong Z et al (2011)^15^ suggesting that bioactive glass has the potential to remineralize artificial carious enamel and dentin.

Thus, the initial carious lesions can be treated non-invasively by remineralization with calcium, phosphate, and fluoride to restore the strength, esthetic appearance and to increase the resistance to future acid challenge.

However, this *in vitro* study had certain limitations like difficulty in simulating the oral environment, lower level of salivary proteins, lack of bacteria in the artificial saliva solution used, control over the salivary flow rate and a harsher acidogenic challenges used in a shorter period of time.

Even though bioactive glass (BAG) has got superior remineralizing potential over CPP-ACPF, ReminPro and fluoridated toothpaste, long-term *in vivo* studies will be needed to prove their beneficial effect for the routine use in the prevention of dental caries.

## CONCLUSION

- SHY-NM, ReminPro and GC Tooth Mousse Plus are effective in remineralizing artificially induced caries lesions.
- The bioactive particles in SHY-NM is superior to the GC Tooth Mousse Plus, ReminPro and fluoridated toothpaste.
